# Dynamic Reprogramming of Immune-Related Signaling During Progression to Enzalutamide Resistance in Prostate Cancer

**DOI:** 10.3390/cancers17193187

**Published:** 2025-09-30

**Authors:** Pengfei Xu, Huan Qu, Joy C. Yang, Fan Wei, Junwei Zhao, Menghuan Tang, Leyi Wang, Christopher Nip, Henson Li, Allen C. Gao, Kit Lam, Marc Dall'Era, Yuanpei Li, Chengfei Liu

**Affiliations:** 1 Department of Urologic Surgery, School of Medicine, University of California, Davis, CA 95817, USA; pfxu@ucdavis.edu (P.X.); hhqu@ucdavis.edu (H.Q.); jcyang@ucdavis.edu (J.C.Y.); fanwei@ucdavis.edu (F.W.); lcdwang@ucdavis.edu (L.W.); christopher.nip@gmail.com (C.N.); hsnli@health.ucdavis.edu (H.L.); acgao@ucdavis.edu (A.C.G.); mdallera@health.ucdavis.edu (M.D.); 2 Department of Biochemistry and Molecular Medicine, School of Medicine, University of California, Davis, CA 95817, USA; juwzhao@health.ucdavis.edu (J.Z.); tmhtang@health.ucdavis.edu (M.T.); kslam@health.ucdavis.edu (K.L.); lypli@health.ucdavis.edu (Y.L.); 3 Graduate Group in Integrative Pathobiology, University of California, Davis, CA 95817, USA; 4 UC Davis Comprehensive Cancer Center, Sacramento, CA 95817, USA

**Keywords:** prostate cancer, immune-related signaling, JAK/STAT, enzalutamide resistance, lineage plasticity

## Abstract

Background: Treatment with androgen receptor (AR) signaling inhibitors, such as enzalutamide, can induce neural lineage plasticity in prostate cancer, potentially progressing to t-NEPC. However, the molecular mechanisms underlying this enzalutamide-driven plasticity, particularly the contribution of immune signaling pathways, remain poorly understood. Methods: We analyzed transcriptomic profiles of patient samples and prostate cancer cell lines to investigate changes in immune signaling pathways. Interferon gamma (IFNγ), interferon alpha (IFNα), and interleukin 6 (IL6)-Janus kinase (JAK)-signal transducer and activator of transcription 3 (STAT3) signaling were assessed in enzalutamide-sensitive and -resistant prostate cancer cells. Functional assays were conducted to examine cell responsiveness to cytokine stimulation and susceptibility to STAT1 inhibition using fludarabine. Results: Immune-related pathways, including IFNγ, IFNα, IL6-JAK-STAT3, and inflammatory responses, were significantly suppressed in NEPC patient samples compared to those with castration-resistant prostate cancer (CRPC). Enzalutamide-resistant and NEPC cells exhibited markedly impaired IFNγ and IL6 signaling. In contrast, early-stage enzalutamide treatment paradoxically enhanced IFNγ and IL6 responsiveness. Transcriptomic profiling revealed coordinated upregulation of E2F target genes and activation of IFNα/IFNγ and JAK/STAT signaling pathways during early treatment. Importantly, these early-stage cells remained highly sensitive to IFNγ and IL6 stimulation and showed increased susceptibility to STAT1 inhibition by fludarabine, a sensitivity that was lost in resistant cells. Conclusions: Early enzalutamide treatment enhances immune responsiveness, while the development of resistance is associated with suppressed immune signaling and increased lineage plasticity. These results suggest a therapeutic window where combining enzalutamide with STAT inhibitors may delay or prevent lineage plasticity and resistance.

## Simple Summary

Enzalutamide treatment can drive lineage plasticity that gives rise to treatment-induced neuroendocrine prostate cancer (t-NEPC), a lethal subtype of prostate cancer. Despite its clinical importance, the immune-related signaling dynamics underlying this enzalutamide-driven plasticity remain poorly understood. This study uncovers a temporal window during early enzalutamide therapy where inflammatory signaling pathways are transiently activated and therapeutically targetable, offering a strategy to delay or prevent neural lineage plasticity transformation and treatment resistance.

## 1. Introduction

Prostate cancer remains the second leading cause of cancer-related death among men in the US [1,2]. While most patients initially respond well to androgen deprivation therapy (ADT), nearly all eventually progress to castration-resistant prostate cancer (CRPC), a lethal form of the disease characterized by continued growth despite low androgen levels [3,4,5]. Advanced CRPC can further evolve into treatment-induced neuroendocrine prostate cancer (t-NEPC), an aggressive variant associated with poor prognosis, lineage plasticity, loss of androgen receptor (AR) signaling dependence, and resistance to conventional therapies [6,7,8,9]. Although de novo NEPC is rare, the widespread use of second-generation AR-targeted therapies such as enzalutamide and abiraterone has led to a rise in the incidence of t-NEPC, underscoring the urgent need to better understand the molecular mechanisms underlying its development [10,11,12].

Increasing evidence suggests that alterations in inflammatory and immune signaling pathways contribute to prostate cancer progression and therapeutic resistance [13,14]. In CRPC, inflammatory cytokines such as interleukin-6 (IL6) and interferons (IFNs) have been implicated in promoting tumor proliferation, survival, and immune evasion [15,16,17]. Chronic inflammation within the tumor microenvironment can foster epithelial-to-mesenchymal transition (EMT), genomic instability, and metastatic potential [18,19,20]. Notably, dysregulation of Janus kinase (JAK)-signal transducer and activator of transcription (STAT) signaling has been shown to drive CRPC progression, promote stemness, facilitate the acquisition of neuroendocrine features, and confer resistance to anti-androgen therapy [21,22,23,24,25]. In addition, impaired inflammatory signaling pathways, including IFNα and IFNγ responses, have been associated with immune escape and lineage plasticity in prostate cancer [26,27,28]. Despite these insights, the dynamic regulation of immune response signaling pathways during the transition from CRPC to neural lineage plasticity and NEPC remains poorly characterized.

AR is a central regulator of prostate cancer biology, and its inhibition profoundly alters cellular signaling networks. AR signaling has been shown to modulate key inflammatory pathways, including the IFNs and IL6-JAK-STAT3 axes [29,30,31]. Enzalutamide, a potent AR antagonist, initially suppresses AR-driven transcription but also triggers compensatory activation of alternative survival pathways, including cytokine signaling cascades [32,33]. Recent studies suggest that AR inhibition can either enhance or suppress IFNs responses depending on cellular context and duration of treatment [34,35]. Moreover, prolonged AR blockade has been implicated in promoting resistance mechanisms through reprogramming of STAT3 activity and inflammatory signaling suppression [31]. However, the precise temporal effects of enzalutamide treatment on IFNs and JAK-STAT pathway activity during the evolution from AR dependence to resistance have not been systematically investigated.

In this study, we comprehensively analyzed the dynamic regulation of immune-related signaling pathways in CRPC and NEPC. Using public transcriptomic datasets and functional assays, we found that inflammation-related pathways, including IFNα, IFNγ, IL6-JAK-STAT3, and general inflammatory responses, were significantly downregulated in NEPC compared to CRPC. We demonstrated that enzalutamide-resistant and NEPC cells exhibit impaired IFNγ and IL6 signaling, while short-term enzalutamide treatment paradoxically enhanced IFNγ and IL6 responsiveness and E2F target gene activation. The early stage of lineage plasticity transition from CRPC to NEPC may require cooperative priming by both the JAK-STAT and E2F pathways. Importantly, early-stage enzalutamide-treated cells remained sensitive to STAT1 inhibition by fludarabine, suggesting an opportunity to intervene before the establishment of full resistance. These findings reveal a potential therapeutic window of inflammatory pathway activation during early AR blockade and propose novel strategies to delay or prevent treatment-induced lineage plasticity in advanced prostate cancer.

## 2. Materials and Methods

### 2.1. Reagents and Cell Culture

C4-2B cells were generously provided and authenticated by Dr. Leland Chung’s laboratory (Cedars-Sinai Medical Center, Los Angeles, CA, USA). C4-2B MDVR (enzalutamide-resistant C4-2B) cells were established by long-term culture of parental C4-2B cells in 20 μM enzalutamide, with parallel cultures of untreated C4-2B cells maintained as controls. C4-2B-1M refers to C4-2B cells exposed to 20 μM enzalutamide for one month. CWR22Rv1 and H660 cells were obtained from the American Type Culture Collection (ATCC).C4-2B, C4-2B MDVR, and CWR22Rv1 cells were maintained in RPMI 1640 medium supplemented with 10% fetal bovine serum (FBS), 100 U/mL penicillin, and 0.1 mg/mL streptomycin. H660 cells were grown in RPMI 1640 containing 5% FBS, 0.005 mg/mL insulin, 0.01 mg/mL transferrin, 30 nM sodium selenite, 10 nM hydrocortisone, 10 nM β-estradiol, and 2 mM L-glutamine. All cultures were kept at 37 °C in a humidified incubator with 5% CO_2_. Enzalutamide (Selleck Chemicals, Houston, TX, USA) was dissolved in dimethyl sulfoxide (DMSO) before use. Recombinant human IL-6 (Sino Biological, Cat# 10098-HNAE, Beijing, China) and IFN-γ (R&D Systems, Cat# 285-IF, Minneapolis, MN, USA) were used for cytokine stimulation as indicated. 

### 2.2. Western Blot Analysis and Antibodies

Whole-cell lysates were prepared and resolved by SDS-PAGE, then transferred onto nitrocellulose membranes. After blocking in 5% non-fat milk in PBS/0.1% Tween-20 for 1 h at room temperature, membranes were incubated overnight at 4 °C with the following primary antibodies: p-STAT1 (#5806, 1:1000, Cell Signaling Technology, Danvers, MA, USA), STAT1 (#sc-417, 1:1000, Santa Cruz Biotechnology, Dallas, TX, USA), p-STAT3 (#9145, 1:1000, Cell Signaling Technology, Danvers, MA, USA), STAT3 (#9132, 1:1000, Cell Signaling Technology, Danvers, MA, USA), cleaved PARP (#5626, 1:1000, Cell Signaling Technology, Danvers, MA, USA), PARP (#9142, 1:1000, Cell Signaling Technology, Danvers, MA, USA), Cyclin A (#4656, 1:1000, Cell Signaling Technology, Danvers, MA, USA), Cyclin D1 (#2978, 1:1000, Cell Signaling Technology, Danvers, MA, USA), Tubulin (T5168, 1:5000, Sigma-Aldrich, St. Louis, MO, USA), Histone H3 (#ab1220, 1:1000, Abcam, Cambridge, UK), HSP90 (#ab13492, 1:1000, Abcam, Cambridge, UK), and GAPDH (#2118, 1:1000, Cell Signaling Technology, Danvers, MA, USA). GAPDH was used as a loading control. After washing, membranes were incubated with HRP-conjugated secondary antibodies (Promega, W4011 and W4021, 1:5000, Madison, WI, USA). Bands were visualized using an enhanced chemiluminescence (ECL) detection system (Millipore, Billerica, MA, USA).

### 2.3. Real-Time Quantitative RT-PCR

Total RNA was isolated using the RNeasy Mini Kit (Qiagen, Hilden, Germany), and reverse transcription was performed according to the manufacturer’s instructions. Quantitative RT-PCR was carried out using SsoFast EvaGreen Supermix (Bio-Rad, Hercules, CA, USA) on a CFX96 Real-Time PCR Detection System (Bio-Rad, Hercules, CA, USA), as previously described [36]. GAPDH served as the internal reference gene. Each reaction was run in triplicate. Primer sequences are listed in Table S1 (in the Supplementary Materials).

### 2.4. Cell Growth Assay

C4-2B, C4-2B cells treated with 20 μM enzalutamide for one month (C4-2B-1M), C4-2B MDVR, or CWR22Rv1 cells were seeded at a density of 1 × 10^4^ cells per well in 12-well plates. After indicated treatments, the number of viable cells was determined by total cell counts.

### 2.5. Clonogenic Assay

C4-2B, C4-2B-1M, and C4-2B-MDVR cells were seeded at a density of 1000 cells per well in 6-well plates and treated as indicated. After 10–14 days, colonies were washed with PBS, fixed, and stained with 0.5% crystal violet in 4% formaldehyde for 30 min. Colony numbers were counted manually.

### 2.6. Luciferase Assay

C4-2B, C4-2B-1M, C4-2B-MDVR, CWR22Rv1, and H660 cells were transfected with the pLucTKS3 luciferase reporter plasmid containing a Stat3-specific response element. Following transfection, cells were stimulated with IL-6 (10 ng/mL) or IFNγ (20 ng/mL) for 48 h. Luciferase activity was measured in cell lysates using the Luciferase Assay System (Promega), as previously described [37]. To account for variability in transfection efficiency, we calculated the relative luciferase activity by comparing the signal from cytokine-stimulated cells with that from unstimulated cells within the same transfection group.

### 2.7. RNA-Seq Data Analysis

Total RNA was isolated from control and IFNγ-treated C4-2B, C4-2B-1M, and C4-2B-MDVR cells using the RNeasy Mini Kit (Qiagen, Hilden, Germany) with on-column DNase digestion. RNA sequencing was performed simultaneously on C4-2B, C4-2B-1M, and C4-2B-MDVR cells. All samples were processed in the same experimental batch to minimize technical variation and ensure direct comparability across the three cell lines. RNA-seq libraries were generated from 1 μg of total RNA using the Illumina TruSeq RNA Sample Kit (v2) and sequenced on the Illumina HiSeq 4000 platform (Illumina, San Diego, CA, USA) to produce paired-end 150 bp reads (~30 million reads per sample). Reads were aligned to the reference genome using HISAT (version 2; Johns Hopkins University, Baltimore, MD, USA), and transcript assembly was performed with StringTie (version 3; Johns Hopkins University, Baltimore, MD, USA). Expression values were calculated as fragments per kilobase of transcript per million mapped reads (FPKM). Differential expression analysis was performed using the TopHat–Cufflinks pipeline.

### 2.8. Gene Set Enrichment Analysis (GSEA)

GSEA was performed with the Broad Institute’s Java application (http://software.broadinstitute.org/gsea/index.jsp, accessed on 24 July 2025) following published protocols [38]. Pathways were considered significantly enriched when the normalized enrichment score (NES) was positive, the nominal *p*-value was < 0.05, and the false discovery rate (FDR) *q*-value was < 0.25.

### 2.9. Datasets and Patients’ Cohort

Clinical and molecular data from the Beltran castration-resistant neuroendocrine prostate cancer cohort were retrieved from cBioPortal for Cancer Genomics (https://www.cbioportal.org/, accessed on 20 March 2024).

### 2.10. Statistical Analysis

RNA sequencing was performed on a single sample for each cell line. All other experiments were conducted with three independent biological replicates (*n* = 3), unless otherwise indicated. Statistical analyses were performed using GraphPad Prism 9.0 (RRID:SCR_002798). Data were summarized as means ± standard deviations (SD) and visualized using appropriate graphical formats. Normality was assessed, and data were transformed as needed. Sample sizes were determined based on statistical power calculations. No data points or samples were excluded. Experiments were not blinded. Comparisons between two groups were performed using two-tailed Student’s *t*-tests, and comparisons among multiple groups were assessed using one-way ANOVA followed by Scheffé’s post hoc test. A *p*-value < 0.05 was considered statistically significant (* *p* < 0.05, ** *p* < 0.01, *** *p* < 0.001, **** *p* < 0.0001; ns: not significant).

## 3. Results

### 3.1. Inflammation-Associated Pathways Are Downregulated in NEPC

**Figure 1:**
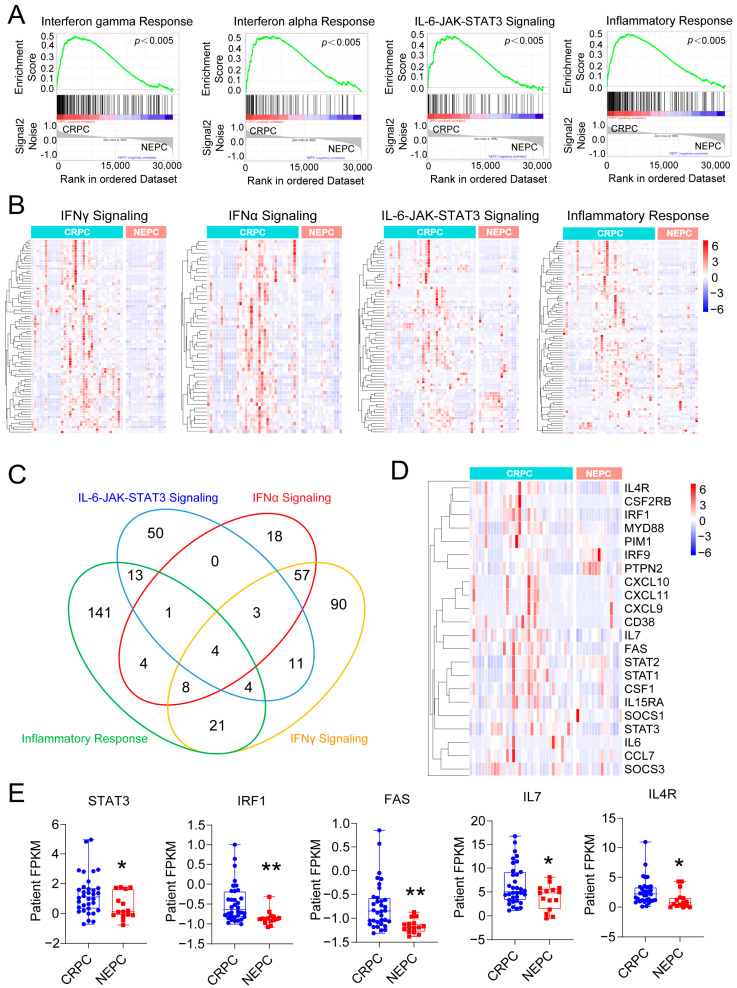
Inflammation-related pathways are downregulated in NEPC. (**A**) Gene set enrichment analysis (GSEA) comparing CRPC and NEPC transcriptomes demonstrates significant downregulation of IFNγ response, IFNα response, IL6-JAK-STAT3 signaling, and inflammatory response pathways in NEPC (FDR < 0.005). (**B**) Heatmaps show decreased expression of genes associated with each pathway in NEPC compared to CRPC. (**C**) Venn diagram illustrating overlap of downregulated genes among the four pathways. (**D**) Heatmap showing the expression of overlapping genes from four inflammatory signaling pathways commonly repressed in NEPC. (**E**) Box plots showing reduced expression of STAT3, IRF1, FAS, IL7, and IL4R in NEPC versus CRPC. Data are presented as mean ± SEM. * *p* < 0.05, ** *p* < 0.01.

GSEA of a public RNA-sequencing dataset [39] revealed that four inflammation-associated pathways, IFNγ, IFNα, IL-6-JAK-STAT3, and the general inflammatory response, were significantly downregulated in NEPC tumors compared to CRPC tumors (Figure 1A). Correspondingly, heatmap analysis showed consistent downregulation of genes within these pathways in NEPC samples (Figure 1B). A Venn diagram comparison of these four pathways identified 23 overlapping genes (Figure 1C), which were further examined via heatmap visualization (Figure 1D). These shared genes were broadly expressed at lower levels in NEPC tumors, with several, including STAT3, IRF1, FAS, IL7, and IL4R, exhibiting statistically significant reductions (Figure 1E). Together, these findings suggest that key inflammation-related signaling pathways, including IFNα, IFNγ, IL-6-JAK-STAT3, and general inflammatory responses, are coordinately suppressed in NEPC.

### 3.2. Enzalutamide-Resistant and NEPC Cells Exhibit Impaired IL-6 Signaling Pathways

**Figure 2:**
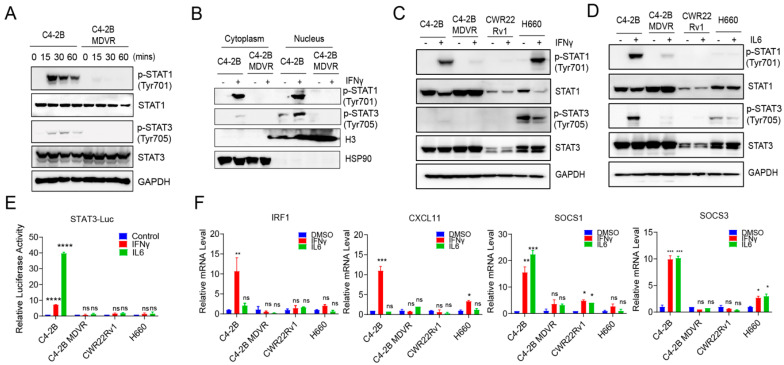
Enzalutamide-resistant and NEPC cells exhibit impaired IL-6 signaling pathways. (**A**) Immunoblot analysis of p-STAT1, total STAT1, p-STAT3, total STAT3, and GAPDH in parental C4-2B and enzalutamide-resistant C4-2B MDVR cells following IFNγ stimulation (20 ng/mL, 0–60 min). (**B**) Cytoplasmic and nuclear fractionation shows impaired nuclear translocation of p-STAT1 and p-STAT3 in C4-2B MDVR compared to C4-2B after IFNγ stimulation (20 ng/mL, 30 min). (**C**) Immunoblot analysis of p-STAT1, total STAT1, p-STAT3, total STAT3, and GAPDH in C4-2B, C4-2B MDVR, CWR22Rv1, and NEPC cell line H660 following IFNγ treatment (20 ng/mL, 30 min). (**D**) Immunoblot analysis of STAT1/3 phosphorylation following IL6 treatment (10 ng/mL, 30 min) in the indicated cell lines. (**E**) Luciferase reporter assay showing reduced p-STAT3 transcriptional activity in resistant and NEPC cells upon IL6 (10 ng/mL, 48 h) or IFNγ stimulation (20 ng/mL, 48 h). (**F**) RT-qPCR analysis of downstream STAT1/3 target genes (*IRF1*, *CXCL11*, *SOCS1*, *SOCS3*) following IL6 (10 ng/mL, 48 h) or IFNγ stimulation (20 ng/mL, 48 h) in the indicated cell lines. Data are shown as mean ± SEM from three biological replicates. * *p* < 0.05, ** *p* < 0.01, *** *p* < 0.001, **** *p* < 0.0001; ns: not significant.

To evaluate the responsiveness of IFNγ and IL-6 signaling across various prostate cancer cell lines, we performed Western blot analysis to assess the phosphorylation of STAT1 (p-STAT1) and STAT3 (p-STAT3) following cytokine stimulation in C4-2B, enzalutamide-resistant C4-2B-MDVR, CWR22Rv1, and H660 cells. As shown in Figure 2A, treatment with IFNγ for 15–60 min led to a marked increase in both p-STAT1 and p-STAT3 levels in C4-2B cells. In contrast, C4-2B-MDVR cells failed to show upregulation of either phospho-protein, suggesting a disruption in the IFNγ signaling cascade. Consistent with this, nuclear-cytoplasmic fractionation demonstrated impaired nuclear translocation of p-STAT1 and p-STAT3 in MDVR cells upon IFNγ stimulation (Figure 2B). We next examined the cytokine responsiveness of CWR22Rv1 and H660 cells. As shown in Figure 2C,D, CWR22Rv1 cells did not exhibit induction of p-STAT1 or p-STAT3 in response to IFNγ or IL-6. In H660 cells, IFNγ stimulation induced p-STAT1 but not p-STAT3, despite a relatively high basal level of p-STAT3 expression. IL-6 stimulation failed to enhance the phosphorylation of either STAT protein in H660 cells (Figure 2C,D). To further confirm these findings, we employed a STAT3-luciferase reporter assay. Both IFNγ and IL-6 induced reporter activity in C4-2B cells, whereas no induction was observed in the other cell lines (Figure 2E). We also analyzed the expression of downstream target genes in the IFNγ and IL-6 signaling pathways. In C4-2B cells, IFNγ robustly induced *IRF1*, *CXCL11*, *SOCS1*, and *SOCS3*, while IL-6 led to the upregulation of *SOCS1* and *SOCS3* only. However, none of these genes were significantly induced by either cytokine in C4-2B-MDVR, CWR22Rv1, or H660 cells (Figure 2F). Notably, although IFNγ-induced phosphorylation of STAT1 was observed in H660 cells, this did not translate into increased expression of downstream targets, further indicating defective transcriptional responses. Similarly, IL-6 signaling remained ineffective in this cell line. Collectively, these results demonstrate that IFNγ and IL-6 signaling pathways are functionally impaired in C4-2B-MDVR, CWR22Rv1, and H660 cells, indicating widespread dysregulation of cytokine responsiveness in advanced or treatment-resistant prostate cancer models.

### 3.3. Enzalutamide Treatment Promotes Interferon Responsiveness at Early Stages

**Figure 3:**
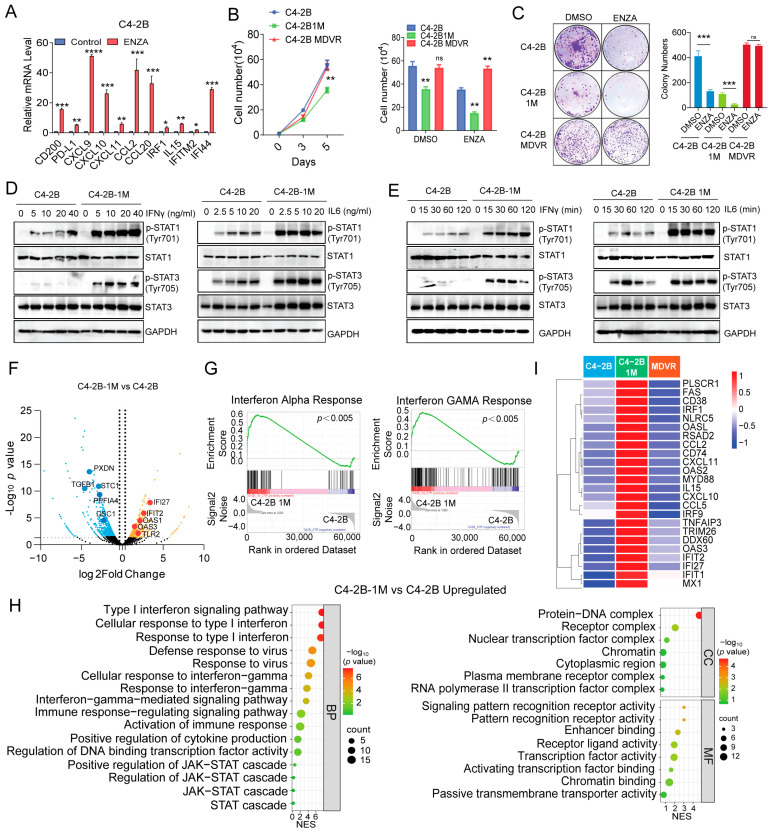
Enzalutamide treatment promotes interferon responsiveness at early stages. (**A**) RT-qPCR analysis shows downregulation of IFNγ target in C4-2B cells after enzalutamide treatment (20 μM) for 2 days. Data are presented as mean ± SEM. (**B**,**C**) Growth curve and colony formation assays show that C4-2B-1M cells proliferate more slowly than both parental C4-2B and resistant C4-2B MDVR cells, and remain sensitive to continued enzalutamide treatment. (**D**) Western blot analysis of p-STAT1 and p-STAT3 levels in C4–2B and C4-2B-1M cells following 1-h treatment with the indicated concentrations of IFNγ or IL-6. (**E**) Western blot analysis of p-STAT1 and p-STAT3 levels in C4-2B and C4-2B-1M cells following treatment with IFNγ (20 ng/mL) or IL-6 (10 ng/mL) for the indicated time points. (**F**) Volcano plot of differentially expressed genes between C4-2B-1M and C4-2B parental cells. (**G**) GO enrichment analysis of upregulated pathways in C4-2B-1M highlights activation of interferon pathways. (**H**) GSEA shows upregulation of interferon-α and interferon-γ response signatures in C4-2B-1M versus parental C4-2B cells. (**I**) Heatmap of selected upregulated genes associated with interferon pathways in C4-2B-1M cells. * *p* < 0.05, ** *p* < 0.01, *** *p* < 0.001, **** *p* < 0.0001, ns: not significant.

To investigate the impact of enzalutamide treatment on IFNγ signaling, we first treated C4-2B cells with enzalutamide for 2 days and assessed the expression of IFNγ target genes by RT-PCR. Interestingly, enzalutamide treatment led to a significant upregulation of IFNγ-responsive genes, including *CD274* (PD-L1), *CXCL10*, *CXCL11*, and *IRF1* (Figure 3A). To examine the effects of prolonged enzalutamide exposure on immune response signaling, we established a C4-2B subline treated with enzalutamide continuously for one month (referred to as C4-2B-1M). Leveraging the enzalutamide-resistant C4-2B-MDVR cells, which we previously identified as having acquired neural lineage plasticity [40], we compared them with the parental C4-2B cells. In contrast, the C4-2B-1M cells exhibited reduced proliferation and retained sensitivity to enzalutamide. (Figure 3B), a finding further supported by clonogenic assay results (Figure 3C). Next, we evaluated cytokine responsiveness in C4-2B-1M cells. Compared to parental C4-2B cells, the C4-2B-1M cells displayed significantly increased phosphorylation of STAT1 and STAT3 following IFNγ and IL-6 stimulation, in a dose- and time-dependent manner (Figure 3D,E), indicating enhanced cytokine sensitivity. To further characterize the molecular changes induced by prolonged enzalutamide treatment, we performed RNA sequencing of C4-2B and C4-2B-1M cells. A total of 375 genes were significantly upregulated, and 987 genes were downregulated in the C4-2B-1M cells. Notably, several interferon-stimulated genes, including *IFI27*, *IFIT2*, *OAS1*, *OAS3*, and *TLR2*, were among the most upregulated genes (Figure 3F).

Gene Ontology (GO) analysis revealed significant enrichment of biological pathways related to type I and type II interferon signaling, as well as antiviral responses, in C4-2B-1M cells (Figure 3G). Consistently, Gene Set Enrichment Analysis (GSEA) confirmed strong enrichment of IFNα and IFNγ signaling pathways in these cells (Figure 3H). Heatmap visualization further highlighted the upregulation of key IFNs-related genes, including *IRF1*, *CXCL10*, *CXCL11*, and *IL15*, in C4-2B-1M cells (Figure 3I). In summary, prolonged enzalutamide treatment enhances the responsiveness of prostate cancer cells to IFNγ and IL-6 stimulation and induces robust activation of interferon signaling pathways. These results suggest that enzalutamide induces an early-stage immune-related reprogramming in prostate cancer cells, potentially sensitizing them to cytokine-mediated signaling and immune modulation.

### 3.4. Early Enzalutamide Treatment Upregulates E2F Target Gene Expression Aligned with JAK/STAT Signaling Activation

**Figure 4:**
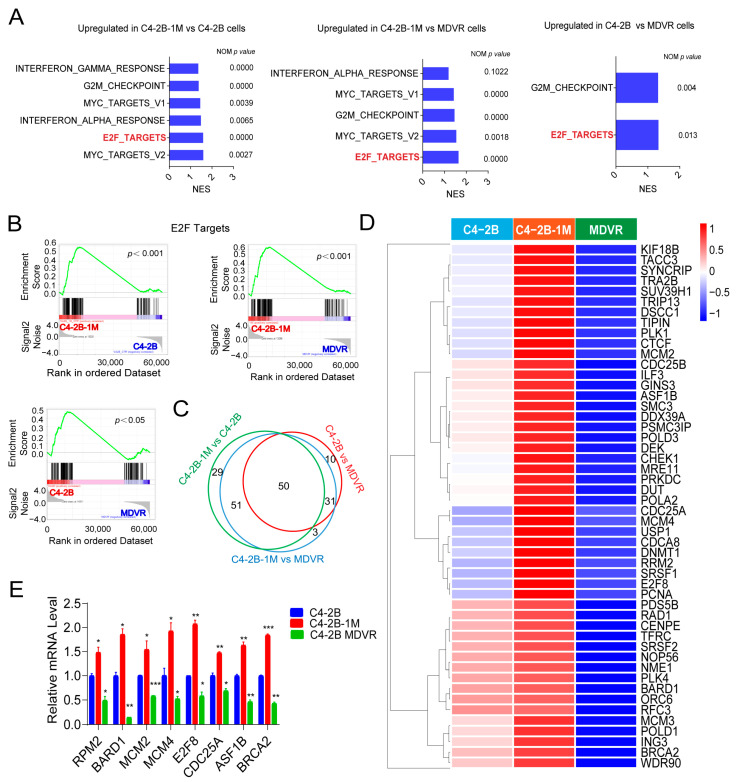
Enzalutamide treatment upregulates E2F target gene expression. (**A**) Selected hallmark pathways enriched in gene expression profiles comparing C4-2B-1M vs. C4-2B, C4-2B vs. MDVR, and C4-2B-1M vs. MDVR cells, highlighting a consistent upregulation of E2F target signatures following short-term enzalutamide exposure. (**B**) GSEA plots showing significant enrichment of E2F target gene sets in C4-2B-1M compared to parental C4-2B, C4-2B compared to MDVR, and C4-2B-1M compared to MDVR cells (*p* < 0.005). (**C**) Venn diagram showing overlapping upregulated E2F target genes across three comparisons (C4-2B-1M vs. C4-2B, C4-2B vs. MDVR, and C4-2B-1M vs. MDVR). (**D**) Heatmap showing expression patterns of commonly upregulated E2F target genes among the three groups. (**E**) RT-qPCR validation of representative E2F target genes in C4-2B, C4-2B-1M, and MDVR cells. Data are presented as mean ± SEM. (* *p* < 0.05, ** *p* < 0.01, *** *p* < 0.001).

To investigate the mechanism underlying the enhanced IFNs responsiveness observed after one month of enzalutamide treatment, we performed RNA sequencing to compare gene expression profiles among C4-2B, C4-2B-1M, and C4-2B MDVR cells. GSEA revealed significant upregulation of IFNγ and IFNα response pathways, as well as pathways related to the G2/M checkpoint, Myc targets, and E2F targets in C4-2B-1M cells compared to both parental C4-2B and MDVR cells. Among these, only E2F target genes were significantly enriched in C4-2B relative to MDVR cells (Figure 4A).

Notably, E2F signaling was most strongly enriched in C4-2B-1M cells, followed by parental C4-2B, and was lowest in MDVR cells (Figure 4B). A Venn diagram analysis identified 81 commonly upregulated E2F target genes in both C4-2B-1M vs. MDVR and C4-2B vs. MDVR comparisons. Additionally, 29 and 10 genes were uniquely upregulated in C4-2B-1M and C4-2B cells, respectively, with only 3 genes uniquely upregulated in C4-2B-1M cells relative to C4-2B. In total, 50 genes were consistently upregulated across all comparisons (Figure 4C). Heatmap analysis confirmed this pattern, showing the highest expression of E2F target genes in C4-2B 1Month cells, intermediate expression in C4-2B, and lowest expression in MDVR cells (Figure 4D).

These transcriptomic findings were further validated by RT-PCR, which demonstrated increased expression of selected E2F target genes in 1Month cells compared to parental C4-2B cells, and markedly reduced expression in MDVR cells (Figure 4E). Together, these results suggest that enzalutamide treatment induces a transient upregulation of E2F target genes during the early phase of exposure, which diminishes with the development of resistance. This transition substantially overlaps with immune-related signaling changes and highlights a potentially critical role for E2F signaling, in collaboration with JAK/STAT signaling, in the adaptive transcriptional response to enzalutamide.

### 3.5. Enzalutamide-Treated Prostate Cancer Cells in the Early Stage Are Highly Responsive to IFNγ

**Figure 5:**
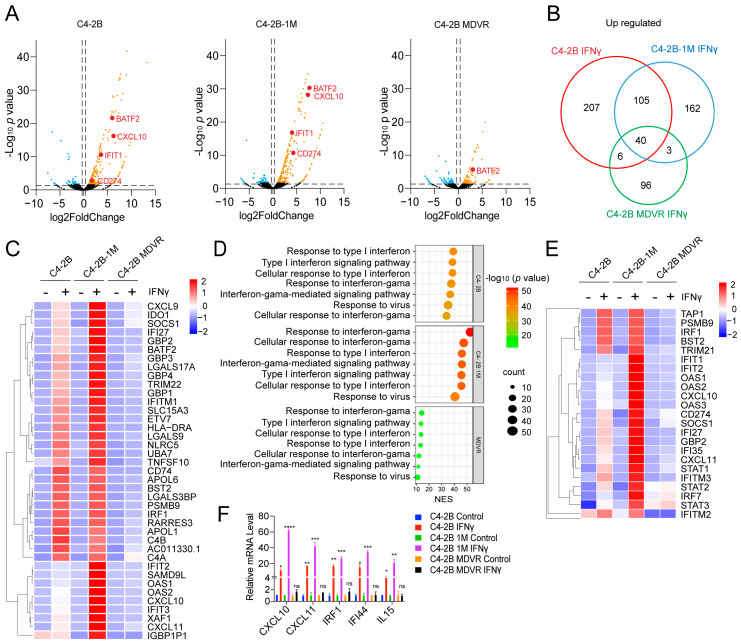
Enzalutamide-treated cells retain IFNγ sensitivity during early treatment. (**A**) Volcano plots showing differential gene expression in C4-2B, C4-2B-1M, and C4-2B MDVR cells after IFNγ stimulation. Key upregulated interferon-stimulated genes such as *CXCL10*, *BATF2*, *CD274*, and *IFIT1* are highlighted. (**B**) Venn diagram comparing significantly upregulated genes following IFNγ treatment in the three cell lines. (**C**) Heatmap showing expression profiles of shared and unique IFNγ-induced genes. (**D**) GO enrichment analysis of IFNγ-induced genes reveals enrichment of type I and II interferon signaling pathways, cellular responses to virus, and inflammatory processes across the groups. (**E**) Heatmaps showing expression of representative IFNγ-responsive genes and key interferon-regulated transcription factors across treatment groups. (**F**) RT-qPCR analysis of IFNγ downstream target gene expression in C4-2B, C4-2B-1M, and C4-2B MDVR cells following 24-h IFNγ treatment. * *p* < 0.05, ** *p* < 0.01, *** *p* < 0.001, **** *p* < 0.0001; ns: not significant.

To compare the transcriptional responses of C4-2B, C4-2B-1M, and C4-2B MDVR cells to IFNγ stimulation, we performed RNA sequencing following IFNγ treatment. As shown in Figure 5A, interferon signaling-related genes such as *BATF2*, *CXCL10*, *IFIT1*, and *CD274* were significantly more upregulated in C4-2B-1M cells compared to parental C4-2B cells, while little to no induction was observed in MDVR cells (Figure 5A). Venn diagram analysis revealed that C4-2B and C4-2B-1M cells exhibited a greater number of upregulated genes in response to IFNγ stimulation than MDVR cells, with 145 genes commonly upregulated in both C4-2B and C4-2B-1M cells. In addition, 207 and 162 genes were uniquely upregulated in C4-2B and C4-2B-1M cells, respectively, whereas only 96 genes were upregulated in MDVR cells. A set of 40 genes was commonly induced across all three cell lines (Figure 5B). Heatmap analysis further demonstrated that IFNγ-induced gene expression was most pronounced in C4-2B-1M cells, moderate in C4-2B cells, and minimal in MDVR cells (Figure 5C). GO enrichment analysis indicated that IFNγ-treated 1 Month cells exhibited strong activation of pathways associated with both type I and type II interferon responses, as well as antiviral defense pathways. These responses were more robust than those observed in parental C4-2B cells and were largely absent in MDVR cells (Figure 5D). A heatmap of representative IFNγ downstream genes further validated this pattern across the three cell lines (Figure 5E). To confirm these transcriptomic findings, we performed RT-PCR analysis of selected IFNγ target genes, including *IRF1*, *CXCL10*, and *CXCL11*. Consistent with the RNA-seq data, 1 Month cells showed higher levels of IFNγ-induced expression compared to C4-2B cells, while MDVR cells showed no significant induction (Figure 5F). In summary, enzalutamide treatment enhances IFNγ signaling during the early stages of therapy, as evidenced by increased expression of interferon-responsive genes. However, this enhancement is lost upon the development of resistance, highlighting dynamic reprogramming of cytokine responsiveness during enzalutamide treatment.

### 3.6. Early-Stage Enzalutamide Exposure Enhances Fludarabine Response in Prostate Cancer Cells

**Figure 6:**
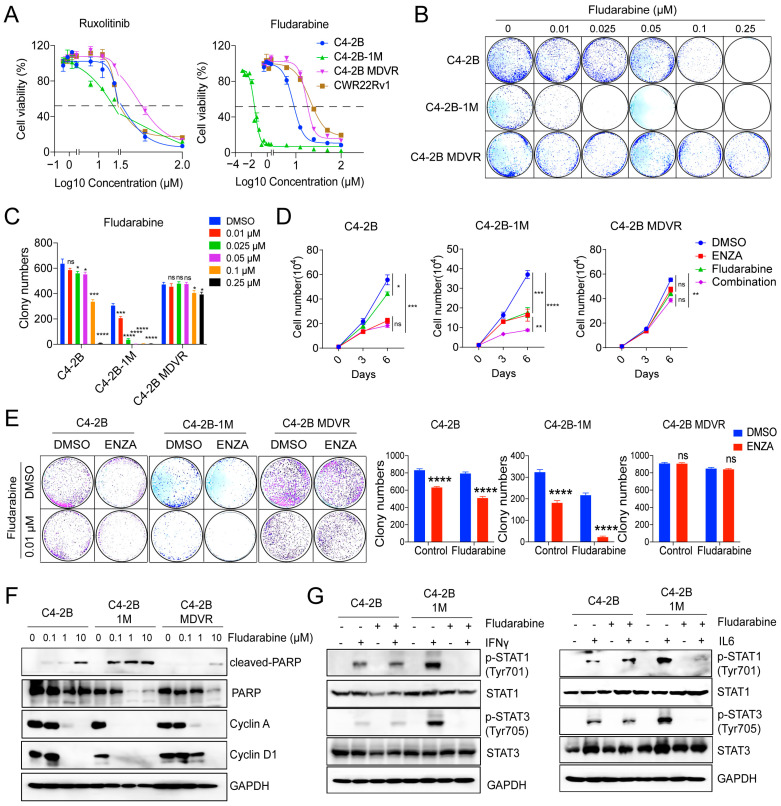
Enzalutamide-treated cells show increased sensitivity to fludarabine at early stages. (**A**) Dose–response curves of Ruxolitinib and Fludarabine in C4-2B, C4-2B-1M, C4-2B MDVR, and CWR22Rv1 cells, assessed by CellTiter-Glo assay to determine cell viability and drug IC50 values. (**B**) Colony formation assay of the indicated cell lines treated with increasing concentrations of Fludarabine. (**C**) Quantification of colony numbers from (**B**). (**D**) Growth curves of C4-2B, C4-2B-1M and C4-2B MDVR cells treated with DMSO, enzalutamide (20 μM), Fludarabine (0.1 μM), or the combination. (**E**) Colony formation assay of C4-2B 1M cells treated with DMSO, enzalutamide (20 μM), Fludarabine (0.01 μM), or the combination. Quantified colony numbers are shown on the right. (**F**) Immunoblot analysis of cleaved PARP, PARP, Cyclin A, and Cyclin D1 in C4-2B, C4-2B-1M, and C4-2B MDVR cells after treatment with Fludarabine. (**G**) Western blot analysis of STAT1 and STAT3 phosphorylation in response to IFNγ (20 ng/mL) with or without Fludarabine (0.1 μM) in C4-2B and C4-2B-1M cells. * *p* < 0.05, ** *p* < 0.01, *** *p* < 0.001, **** *p* < 0.0001; ns: not significant.

To determine whether enzalutamide treatment alters the sensitivity of prostate cancer cells to JAK-STAT pathway inhibition, we treated C4-2B, C4-2B-1M, and C4-2B MDVR cells with the JAK1/2 inhibitor Ruxolitinib and the STAT1 inhibitor Fludarabine. As shown in Figure 6A, the IC50 values for Ruxolitinib were similar across all three cell lines. In contrast, C4-2B-1M cells displayed markedly increased sensitivity to Fludarabine (Figure 6A). This enhanced sensitivity was further confirmed by clonogenic assays, which demonstrated a significantly greater inhibitory effect of Fludarabine in C4-2B-1M cells compared to the other cell lines (Figure 6B).

We next assessed the combined effect of Fludarabine and enzalutamide in C4-2B-1M cells. Both agents, whether administered individually or in combination, significantly suppressed cell proliferation and colony formation (Figure 6C–E), suggesting potential therapeutic synergy. To further investigate the mechanism of action, we examined the effects of Fludarabine on key cell cycle regulators. In C4-2B-1M cells, Fludarabine at a low concentration (0.1 μM) induced robust cleavage of PARP and downregulation of Cyclin A and Cyclin D1. In contrast, C4-2B parental cells required higher doses to elicit similar effects, and MDVR cells showed minimal response even at elevated concentrations (Figure 6F). We also examined the impact of Fludarabine on IFNγ and IL-6 signaling. Compared to C4-2B parental cells, Fludarabine treatment more effectively suppressed IFNγ- and IL-6-induced phosphorylation of STAT1 and STAT3 in C4-2B-1M cells (Figure 6G), indicating that enhanced cytokine responsiveness in 1 Month cells may contribute to their increased vulnerability to STAT1 inhibition. In summary, enzalutamide treatment sensitizes prostate cancer cells, particularly during the early phase of exposure, to STAT1 inhibition. These findings highlight the potential therapeutic value of early intervention with fludarabine in enzalutamide-treated prostate tumors.

## 4. Discussion

In this study, we investigated the dynamic regulation of inflammatory signaling pathways during prostate cancer progression and the development of therapeutic resistance. Our findings reveal that both enzalutamide resistance and neuroendocrine differentiation are associated with impaired activation of the IFNγ and IL-6-JAK-STAT3 signaling pathways. While short-term enzalutamide treatment enhanced cellular responsiveness to IFNγ and IL-6 and increased sensitivity to STAT1 inhibition, prolonged treatment and resistance were accompanied by marked suppression of immune-related signaling. These results suggest that inflammatory and immune signaling pathways are not static but are actively reprogrammed during disease progression under therapeutic pressure. Mechanistically, we found that resistant, late lineage-plastic or NEPC cells fail to activate key downstream effectors such as p-STAT3 in response to cytokine stimulation. This is accompanied by impaired nuclear translocation and reduced transcriptional activation of interferon-stimulated genes. Such defects likely contribute to immune evasion and therapeutic resistance, particularly in the context of chronic AR-targeted therapy.

Previous studies have shown that AR signaling exerts broad regulatory effects on immune response pathways in prostate cancer [41,42]. AR has been reported to suppress the expression of interferon-stimulated genes and key immune regulators [43,44,45]. Enzalutamide, as an AR signaling inhibitor, can transiently relieve this suppression, resulting in enhanced IFNγ responses and upregulation of inflammatory mediators such as IL5, IL10, and tumor necrosis factor alpha (TNFα) and the chemokine macrophage inflammatory protein 1 alpha (MIP-1α)/chemokine (C-C motif) ligand 3 (CCL3) [46,47,48]. However, chronic exposure to enzalutamide has been associated with the development of an immunosuppressive tumor microenvironment, characterized by decreased antigen presentation and loss of inflammatory signaling [49,50]. Our findings support and extend these observations by showing that enzalutamide initially activates, but later suppresses, key inflammatory pathways as resistance develops. Notably, we demonstrate that short-term enzalutamide treatment (prior to the onset of resistance) leads to a markedly different cellular state. C4-2B cells treated with enzalutamide for one month (C4-2B-1M) exhibited enhanced responsiveness to IFNγ and IL-6, as evidenced by increased phosphorylation of STAT1 and STAT3, robust induction of multiple interferon-stimulated genes, and significant enrichment of both type I and type II interferon response pathways. These results suggest that enzalutamide transiently reprograms AR-dependent prostate cancer cells to adopt a more immunologically active phenotype, potentially creating a therapeutic window during which cytokine-based or immunomodulatory interventions may be particularly effective.

We further observed that activation of the E2F signaling pathway occurs early during enzalutamide treatment, coinciding with enhanced IFNγ responsiveness. E2F transcription factors, particularly E2F1, have been implicated in regulating immune responses by modulating the expression of type I and II interferon-related genes [51,52]. In various cancers, dysregulation of E2F signaling has been linked to alterations in cytokine production and immune evasion [53,54]. Our data suggest that transient activation of E2F signaling following AR blockade may sensitize prostate cancer cells to IFNγ and IL-6 stimulation. However, as resistance develops, this responsiveness diminishes, likely due to further alterations in cell cycle control and immune regulatory pathways. Transcriptomic profiling revealed that the enhanced cytokine responsiveness induced by enzalutamide is accompanied by upregulation of E2F target genes, supporting a role for E2F in priming cells for interferon pathway activation. This elevated inflammatory signaling appears to be transient, potentially curtailed by loss of E2F activation, chromatin remodeling, or negative feedback mechanisms that restrict cytokine signaling during the transition to drug-resistant states.

Transition from CRPC to NEPC represents a major clinical challenge and is accompanied by profound reprogramming of signaling networks. Previous studies have shown that NEPC is characterized by reduced immune cell infiltration, downregulation of MHC class I molecules, and diminished cytokine signaling. The loss of inflammatory pathways has been implicated in promoting lineage plasticity and facilitating immune evasion during neuroendocrine transdifferentiation [26,55]. In line with these observations, our study demonstrates that NEPC cells, such as H660, display defective STAT3 activation and are unresponsive to inflammatory stimuli, including IFNγ and IL-6. Despite elevated basal levels of p-STAT3, H660 cells fail to upregulate downstream target genes upon cytokine stimulation, indicating a decoupling between upstream signaling and transcriptional response. These findings further support the notion that suppression of inflammatory signaling pathways contributes to NEPC pathogenesis and the establishment of an immune-evasive phenotype.

Although previous studies have reported immune-related signaling alterations in enzalutamide-resistant prostate tumors [23,50,56,57], our findings highlight the early stages of resistance acquisition, revealing a transient enhancement of cytokine signaling that has not been fully appreciated. Incorporating additional public datasets [58,59], together with immune-cell deconvolution analyses (e.g., CIBERSORT), could further delineate the dynamic changes in tumor-infiltrating immune populations and strengthen understanding of immune regulation during the transition to drug resistance. Our study reveals that short-term enzalutamide treatment enhances interferon and IL-6 signaling in prostate cancer cells, creating a transient window of immunologic vulnerability that may be therapeutically exploitable. Specifically, C4-2B-1M cells exhibited sustained IFNγ responsiveness and increased sensitivity to the STAT1 inhibitor fludarabine, a purine analog known to suppress STAT1-dependent transcription [60]. It is important to note that fludarabine also exerts broad cytotoxic effects as a nucleoside analog, and thus its activity in our models may reflect a combination of STAT1 inhibition and general cytotoxicity. This effect was not observed in enzalutamide-resistant MDVR cells, highlighting the dynamic reprogramming of cytokine signaling and therapeutic susceptibility during the course of enzalutamide treatment. Although fludarabine is primarily used in hematologic malignancies, emerging evidence suggests it can also modulate immune pathways in solid tumors [61]. In this study, fludarabine was used as a preclinical tool to probe STAT1-dependent signaling; clinical translation would require identification of STAT1- or STAT3-targeted agents with appropriate safety and pharmacologic profiles for prostate cancer patients. Our findings raise the possibility that combining enzalutamide with STAT1- or STAT3-targeted agents early in treatment could enhance efficacy and delay resistance. As enzalutamide treatment progresses, prostate cancer cells undergo adaptive changes that suppress cytokine signaling and facilitate immune escape, underscoring the importance of treatment timing. Collectively, these results support a model in which rational integration of AR-targeted therapy with immunomodulatory agents, particularly during the early treatment phase, may offer a promising strategy to improve outcomes in advanced prostate cancer.

## 5. Conclusions

This study demonstrates that inflammatory and immune signaling pathways in prostate cancer are dynamically reprogrammed during progression and therapeutic resistance. Short-term enzalutamide treatment transiently enhances responsiveness to IFNγ and IL-6, accompanied by increased STAT activation, interferon-stimulated gene expression, and E2F-driven transcriptional priming. This creates a temporary window of immunologic vulnerability in which cytokine-based or immunomodulatory interventions may be particularly effective. However, chronic exposure and resistance are associated with marked suppression of inflammatory signaling, impaired STAT3 activation, and the emergence of immune-evasive, lineage-plastic or neuroendocrine phenotypes. These findings highlight the importance of treatment timing and suggest that rational integration of AR-targeted therapies with agents modulating STAT or cytokine pathways during the early treatment phase may improve therapeutic efficacy and delay resistance in advanced prostate cancer.

## Data Availability

The raw data generated for this study will be made available by the corresponding author upon request.

## Supplementary Materials

The following supporting information can be downloaded at: https://www.mdpi.com/article/10.3390/cancers17193187/s1, Table S1: Primer list for q-PCR.

## Abbreviations

The following abbreviations are used in this manuscript:

AR	Androgen Receptor
ADT	Androgen Deprivation Therapy
CRPC	Castration Resistant Prostate Cancer
tNEPC	Treatment-Induced Neuroendocrine Prostate Cancer
IFNγ	Interferon gamma
IFNα	Interferon alpha
IL6	Interleukin 6
JAK	Janus kinase
STAT3	Signal Transducer and Activator of Transcription 3
STAT1	Signal Transducer and Activator of Transcription 1
EMT	Epithelial to Mesenchymal Transition
TNFα	Tumor Necrosis Factor alpha
MIP1α	Macrophage Inflammatory Protein 1 alpha
CCL3	Chemokine (C C motif) ligand 3
DMSO	Dimethyl sulfoxide
FBS	Fetal Bovine Serum
PBS	Phosphate-Buffered Saline
SDS PAGE	Sodium Dodecyl Sulfate Polyacrylamide Gel Electrophoresis
pSTAT1	Phosphorylated STAT1
pSTAT3	Phosphorylated STAT3
PARP	Poly (ADP ribose) polymerase
RT-PCR	Reverse Transcription Polymerase Chain Reaction
qPCR	Quantitative Polymerase Chain Reaction
RNAseq	RNA sequencing
FPKM	Fragments Per Kilobase of transcript per Million mapped reads
GSEA	Gene Set Enrichment Analysis
NES	Normalized Enrichment Score
FDR	False Discovery Rate
GO	Gene Ontology
ATCC	American Type Culture Collection
ANOVA	Analysis of Variance
MHC	Major Histocompatibility Complex
G2M	Gap 2 Mitosis phase (cell cycle)
Myc	Myelocytomatosis oncogene
E2F	E2F Transcription Factor
PDL1	Programmed Death Ligand 1 (also known as CD274)
SOCS1	Suppressor of Cytokine Signaling 1
SOCS3	Suppressor of Cytokine Signaling 3
IRF1	Interferon Regulatory Factor 1
CXCL10	C X C Motif Chemokine Ligand 10
CXCL11	C X C Motif Chemokine Ligand 11
IFI27	Interferon Alpha Inducible Protein 27
IFIT2	Interferon Induced Protein with Tetratricopeptide Repeats 2
OAS1	2 5 Oligoadenylate Synthetase 1
OAS3	2 5 Oligoadenylate Synthetase 3
TLR2	Toll Like Receptor 2
BATF2	Basic Leucine Zipper ATF Like Transcription Factor 2
IFIT1	Interferon Induced Protein with Tetratricopeptide Repeats 1
CD274	Cluster of Differentiation 274 (also known as PDL1)
RPMI	Roswell Park Memorial Institute (refers to culture medium)
ECL	Enhanced Chemiluminescence
HRP	Horseradish Peroxidase
GAPDH	Glyceraldehyde 3 Phosphate Dehydrogenase
HSP90	Heat Shock Protein 90
cBioPortal	cBio Portal for Cancer Genomics
